# How Socio-Economic Drivers Explain Landscape Soil Erosion Regulation Services in Polish Catchments

**DOI:** 10.3390/ijerph19042372

**Published:** 2022-02-18

**Authors:** Mustafa Nur Istanbuly, Josef Krása, Bahman Jabbarian Amiri

**Affiliations:** 1 Department of Environmental Science, Faculty of Natural Resources, University of Tehran, Chamran Blvd., Karaj 3158777878, Iran; istanbuly@ut.ac.ir; 2 Department of Landscape Water Conservation, Faculty of Civil Engineering, Czech Technical University in Prague, Thakurova 7, 16629 Prague, Czech Republic; josef.krasa@cvut.cz; 3 Department of Regional Economics and the Environment, Faculty of Economics and Sociology, University of Łódź, 90-255 Lodz, Poland

**Keywords:** landscape, ecosystem services, soil erosion regulation, area-weighted average income per capita, area-weighted average GDP, HDI

## Abstract

Most studies that address the relationship between socio-economic characteristics and soil erosion focus on the effects of soil erosion on socio-economic conditions at different levels, from global to smallholder. Few, if any, efforts are made to address the influence of socio-economic variables on the soil erosion rate as an indicator of landscape degradation. The present study was carried out using spatial data from 402 catchments that cover Poland, to find out how socio-economic variables, which include area-weighted average income per capita (PLN km^−2^), area-weighted average gross domestic product (PLN km^−2^), population density (person km^−2^), and human development index can drive the soil erosion rate (kg ha^−1^ yr^−1^), along with annual precipitation, soil and geomorphological variables that include soil organic carbon content, soil water content, clay ratio, stream gradient, and terrain slope. The results showed that the soil erosion rate is indirectly driven by the socio-economic variables in the study catchments, as it is alleviated by increasing population density, the area-weighted average gross domestic product, and the human development index. Furthermore, analyzing the incremental relationship between soil erosion rate and the area-weighted average of socio-economic variables revealed that no uniform change can be observed in the relationship between the area-weighted average socio-economic variables and soil erosion in the study catchments.

## 1. Introduction

Among the various landscape degradation processes, soil erosion is recognized as a major environmental issue that causes the loss of topsoil and nutrients, and reduces soil fertility [1]. The effects of soil erosion begin with changes in the physical, chemical, and biological properties of the soil, and gradually result in a decrease in soil productivity capacity [2].

The main drivers of water erosion include geomorphological factors such as the physical characteristics of a catchment, steep slopes, the density of the drainage network, and the gradient of the stream [3,4], climatic factors, such as rainfall, rainfall intensity, the number of rainy days per year and climate types [4,5], along with soil factors and human activities, such as deforestation, overgrazing, and intensive agriculture [3,6], all of which affect the soil erosion process.

Soil erosion is not only a physical and economic problem of landscape degradation and loss of natural capital [7], but soil degradation is also a global challenge for sustainable agriculture [7,8]. Several studies (e.g., see [9,10,11,12]) show that soil erosion is detrimental to global food production and has led to a reduction in agricultural production by 33.7 million tons [9]. Furthermore, it has a significant negative influence on food security by reducing global agricultural production and, in turn, increasing the prices of agricultural products by 0.4% to 3.5% in the world [9].

Economic losses of soil erosion can be determined at the farm level [8,13,14], the catchment level [15,16,17], or even nationally [7]. Soil erosion and the consequent land losses put more pressure on resources, such as natural land cover (forests, grasslands, and even water bodies) and uncontrolled groundwater extraction. Soil erosion costs can generally be divided into on-site and off-site costs. The on-site damage includes nutrient and yield losses, land depletion, and biological losses. Meanwhile, the off-site damage includes, but is not limited to, sedimentation [2,18], floods [2,18,19], infrastructure degradation [2,19], decline in agricultural production [11,12,19], food price increases [2,19,20], change in land use/land cover [14,21], water and biodiversity losses [9], natural disasters [22,23], and global warming [24,25].

Landscape degradation in general and soil erosion, in particular, are associated with many negative social, political, and economic effects [2]. Many studies (see, e.g., [7,8,9,19,20,21]) describe the mechanism of soil erosion and its economic and social effects. Pimentel et al. [26] showed that the cost of soil erosion in the United States, for example, exceeds US $16 billion per year.

Sun et al. [20] indicated that soil erosion and socio-economic variables interact with each other. Although soil erosion affects the social and economic development of a given region, socio-economic development, in turn, causes a sharp increase in the demand of inhabitants for more exploitation of natural resources, which in turn increases the rate of water and soil losses. On the other hand, socio-economic development promotes people’s understanding of soil erosion, allowing them to consciously change their production systems and lifestyle and allocate sufficient funds to conserve soil and water.

There are several studies (see, e.g., [27,28,29]) that revealed that increasing urbanization increasingly occurs at the expense of rural life. Agriculture-related practices such as farming and ranching are then substituted by non-agricultural activities. This change in landscape composition, in turn, reduces the extent of land degradation, which is later accompanied by a reduction in soil erosion and a favorable environment for the restoration of natural ecosystems.

Wang et al. [21] pointed out that soil erosion occurs after a period of rapid economic development and urbanization. However, the increase in GDP, which is usually measured on a national scale, may be due, in part, to ecological pressures on land resources, indirectly reducing the risk of soil erosion for humans. [19] estimated that 12 million hectares of agricultural areas in the EU suffer severe soil erosion, which is equal to 0.43% of their annual crop production. It is also estimated that the resulting loss in agricultural productivity is around €1.25 billion annually. 

Many studies (e.g., see [2,9,12,13]) have examined the relationship between soil erosion and various socio-economic variables to determine the effects that soil erosion may have on socio-economic conditions on macro and microeconomic scales [9]. In Malawi, for example, the loss of topsoil due to soil erosion can be regarded as a serious threat to the economic development of the country because approximately 26% of GDP is generated by the value added of the agricultural sector and the proportion of the rural population of the country is greater than 80% [12]. These values for Poland are 2.5% [30] and 39.96%, respectively [31].

Eaton [8] and Barbier [13] indicated that household income as one of the microeconomic variables could be an important factor that affects soil erosion. Some farming households may decide to spend money on soil conservation practices based on their income, while others may assume there is no need to invest, relying instead on a short-term economic analysis approach [13]. A challenge that may arise is how to achieve a compromise between minimizing soil erosion and maximizing income as two competing objectives [32]. The approach to solving this problem could be to define the optimal level of soil conservation, in which the marginal benefits of additional soil protection equate only to its costs [13].

Gocić et al. [16], who focused on the relationship between demographic changes and soil erosion, indicated that demographic changes can be counted as a factor that causes an indirect change in soil erosion rate; they can play a role in controlling the soil erosion rate through a change in land use/land cover. They also showed that the amount of soil erosion in the Jablanica river catchment in Serbia was 654.41 (m^3^ km^−2^ yr^−1^) in 1971; however, it decreased to 472.03 (m^3^ km^−2^ yr^−1^) in 2016. Bilsborrow [33] revealed that those countries that experience high growth rates in their rural populations face widespread landscape degradation and soil erosion due to the need for more arable land to meet the needs of their local people. It is usually associated with increased deforestation and degradation of other natural land covers, such as forests and grassland.

It should be noted that Poland has experienced significant changes in the distribution of the urban-rural population in recent decades, with the share of the rural population decreasing from 63.1% in 1950 to 39% in 2009 [34]. Biegańska and Szymańska [35] showed that migration continued until 2011, and these population dynamics have increased the population density in cities and suburbs so that the source of livelihood of migrant households is no longer only agriculture; it is also dependent on the non-agricultural sources of income.

Rivlin [36] showed that human development leads to landscape degradation and soil erosion, and the extent of the resulting degradation is highly dependent on natural resource management policies. Environmental degradation, as evidenced by indicators such as carbon dioxide emissions, deforestation, freshwater extraction, and soil erosion, is jeopardizing human development achievements. Furthermore, as environmental indicators show, current progress and development are detrimental to the next generation [37].

A crucial question that may arise is whether socio-economic conditions affect soil erosion and drive landscape degradation in general, and soil erosion in particular, or alleviate the extent of landscape degradation, in turn, reducing the amount of soil erosion. These are the questions we sought to answer through the present study.

## 2. Materials and Methods

### 2.1. Study Area

**Figure 1:**
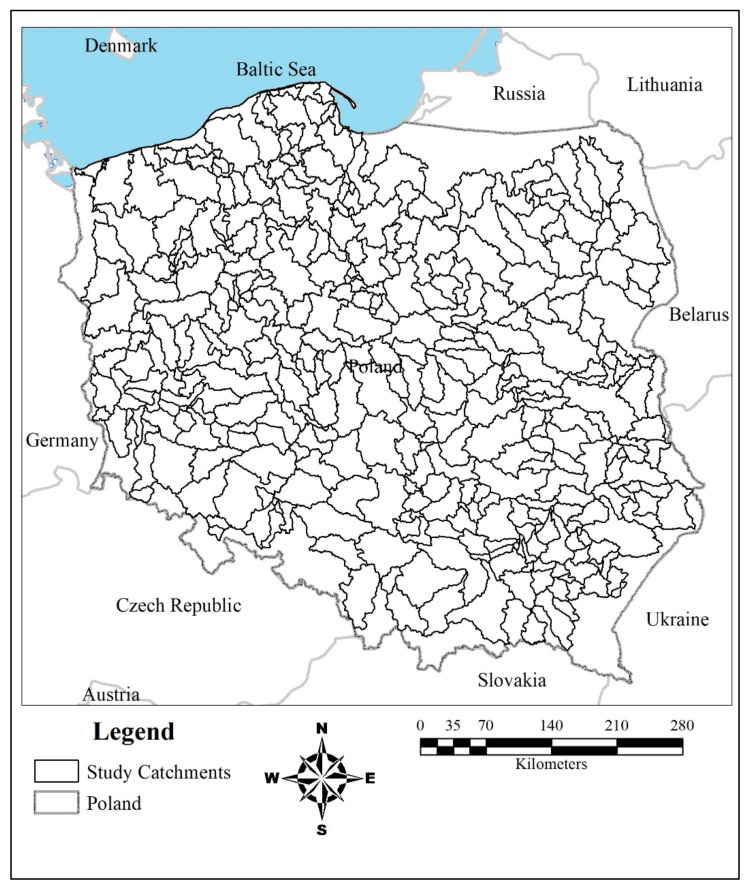
The geographical position of the study catchments in Poland.

The study area consists of 402 catchments located within Poland, ranging between 0.23 km^2^ and 2758.03 km^2^ (Table 1). These catchments are distributed in elevation between −3 m and 658 m above sea level, mean terrain slope 12.26 ± 10.01%, annual precipitation 580.65 ± 53 (mm yr^−1^), and mean stream gradient 15.51 (dm km^−1^). The mean population density is 129.17 (person km^−2^), the area-weighted average income per capita is 4.25 (PLN km^−2^), the area-weighted average GDP is 44.35 (PLN km^−2^), and HDI is 0.85 ± 0.01 (Figure 1).

### 2.2. Data Description

The digital map of the study catchments, along with annual precipitation (mm yr^−1^), terrain slope (degrees), stream gradient (dm km^−1^), soil organic carbon content (ton ha^−1^), clay ratio, soil water content (%) and the soil erosion rate (kg ha^−1^ yr^−1^) were acquired from [38] as the source of the data set for the present study. All transboundary catchments have been withdrawn from the data set due to the objective of the study. Socio-economic data were obtained from the [39].

The data set of the study catchments (402, in total) was then randomly divided in proportion (70–30%) into two sub-data sets. Of these, 284 catchments were used for the modeling task and 118 catchments were used to validate the developed models, with the aim of addressing the extent to which they are influenced by the drawbacks originating from the uncertainty.

### 2.3. Methods

The spatial data were transformed into a common digital format, then co-registered with ETRS89 Poland CS92, because they were obtained from various sources. The clay ratio [4] was calculated by the sum of the percentages of sand and silt divided by the percentage of clay. 

Socio-economic data, including population, income per capita and GDP, were obtained from the [39]. All socio-economic data (GDP, income per capita, population), which are based on the Polish administrative boundaries on the county scale, were then transformed into the 402 catchment boundaries (Figure 1) using the area-weighted average technique. 

#### 2.3.1. Modeling

A step-by-step regression model was applied to model the relationship between soil erosion as the dependent variable, soil and geomorphological variables, and socio-economic indicators (population, area- weighted average income per capita, area- weighted average gross domestic product, and human development index) along with annual precipitation as the independent variables. Linear, logarithmic, exponential and power regression models were fitted to find out which one of these model structures could more appropriately explain the relationship between soil erosion and three explanatory factors, that is, socio-economic indicators, soil variables, along with geomorphological variables.

Moreover, variation inflation factors were calculated and examined for each of the model variables to ensure that the models have no multicollinearity issues [40,41]. Soil-erosion models were then evaluated by depicting the observed values against the predicted values [42]. All statistical analyzes and spatial calculations were performed using IBM SPSS for Windows, Release 26, STATISTICA 12, and ArcMap 10.5, respectively. Screening the models developed and selecting the most appropriate model is vital in any modeling work because it reduces the number of models that need to be addressed further in the next steps. It was conducted using inter-model comparison techniques, which are described below.

#### 2.3.2. Inter-Model Comparison

The most appropriate model was selected by applying the Akaike information criterion. The Kulbakk–Leibner data loss index and the maximum likelihood association are shown by this criterion [43,44]. The Akaike information criterion is calculated using Equation (1) [43,45].

(1) $$\mathrm{AIC}\;=\;n\left(\log\frac{\mathrm{RSS}}{n}\right)+2K$$

where AIC is the value of the Akaike information criterion, RSS is the residual sum of squares, K is the number of model variables, including the distance variable from the origin of the model, and *n* is the number of samples (observed or measured).

It is crucial to know to what extent the model response can vary under different conditions and, accordingly, to determine the degree of importance of the model variables. This important work is performed by analyzing the sensitivity of the desired model using the conditional sensitivity analysis method as described by [46].

## 3. Results and Discussion

### 3.1. Results of Modeling

Soil-related variables (clay ratio, soil organic carbon content, soil water content), geomorphological variables (terrain slope, stream gradient) along with annual precipitation, and socio-economic variables (population density (person km^−2^), area-weighted average GDP (PLN km^−2^), area-weighted average income per capita (PLN km^−2^), and HDI) as independent variables and soil erosion rate (kg ha^−1^ yr^−1^) as dependent variable were fitted using a stepwise approach for linear, exponential, logarithmic, and power structures. Equations (2)–(5) show that between 64% and 79% of changes in soil erosion can be achieved with socio-economic variables and a set of soil factors in the study catchments. The statistics of the coefficient of determination, significant at *p* < 0.05, together with additional statistics, are given in Table 2.

(2) $$E=2410.252-8.366\left(\mathrm{SOC}\right)+10.741\left(\mathrm{Slp}\right)-19.547\left(\mathrm{CR}\right)-0.461\left(\mathrm{Stg}\right)-0.538\left(\mathrm{GDP}\right)-2039.122\left(\mathrm{HDI}\right)$$

(3) $$\mathrm{Ln}\left(E\right)=11.814-0.035\left(\mathrm{SOC}\right)-0.108\left(\mathrm{CR}\right)+0.019\left(\mathrm{Slp}\right)-0.001\left(\mathrm{PoD}\right)-5.138\left(\mathrm{HDI}\right)$$

(4) $$\mathrm{Ln}\left(E\right)=11.981-1.662\mathrm{Ln}\left(\mathrm{SOC}\right)-0.915\mathrm{Ln}\left(\mathrm{CR}\right)+0.290\mathrm{Ln}\left(\mathrm{Stg}\right)-5.485\mathrm{Ln}\left(\mathrm{HDI}\right)-0.133\mathrm{Ln}\left(\mathrm{GDP}\right)$$

(5) $$E=1828.741-388.269\mathrm{Ln}\left(\mathrm{SOC}\right)+127.096\mathrm{Ln}\left(\mathrm{Slp}\right)-237.037\mathrm{Ln}\left(\mathrm{CR}\right)-50.363\mathrm{Ln}\left(\mathrm{GDP}\right)-1413.787\mathrm{Ln}\left(\mathrm{HDI}\right)$$

where; E, Soil erosion (kg ha^−1^ yr^−1^); SOC, Soil organic carbon content (ton ha^−1^); Slp, Terrain slope (degree); CR, Clay ratio; Stg, Stream gradient (dm km^−1^); GDP, Area-weighted average gross domestic product (PLN km^−2^); HDI, Human Development Index; PoD, Population density (person km^−2^).

Equations (2)–(5) show an indirect relationship between the values of socio-economic variables (including population density, area-weighted average GDP, and human development index) and soil erosion rates on the national scale. Consequently, if the values of population density, area-weighted average GDP, and HDI increase, the soil erosion rate will decrease in the study catchments. A negative relationship between the human population and the rate of soil erosion has also been indicated by [16], which is consistent with the results of this study. However, the findings of [9] showed that food security is endangered by soil erosion on a global scale as the human population increases.

Gocić et al. [19] noted that the inverse relationship between human population and soil erosion rate is influenced by direct factors such as land management, the development of soil erosion control structures, and the change in land use/land cover. It is also influenced by indirect factors that result from leaving and/or stopping farming practices, which usually occurs because of rural–urban area migration for economic reasons. Agricultural land that is no longer used or cultivated is abandoned and, over time, becomes land covered with grass and natural vegetation. This decreases the share of arable land in the composition of the landscape and ultimately transforms abandoned arable land into other natural land covers, such as grasslands, pastures, or even forest ecosystems. This occurs within the framework and timeline of a natural process called ecological succession.

Bilsborrow [33] indicated that the proportion of agricultural land in the landscape composition increases more in countries with relatively high growth in rural population. Therefore, this change in landscape composition is accompanied by increased deforestation and degradation of lands covered by natural vegetation. Land degradation and soil erosion could hence be accelerated by an increase in rural population. 

On the other hand, an increased rural population and their migration to cities and urban areas can not only reduce the pressure on existing agricultural land, but also reduce more land requirements for agricultural practices. Therefore, it will reduce the rate of transformation of natural land (forests and grasslands) into agricultural land, thus reducing land degradation in general and associated soil erosion in particular. Increasing migrating population to cities and urban areas may result in contributing to non-agricultural incomes in GDP and HDI. Furthermore, a decrease in the area of permeable land in cities and urban areas due to the construction of housing and public facilities can lead to a decrease in the amount of soil erosion. 

In this regard, Okólski and Topińska [34] found that Poland has also experienced some major changes in the distribution of the urban–rural population. The share of the rural population decreased from 63.1% in 1950 to 39.0% in 2009, mainly due to the large population migration from rural to urban areas. Migration from rural to urban areas continued until the 2000s, causing increased population density in cities and urban areas. The main source of income for a large proportion of the migrated families was a source other than agricultural activity [35]. Biegańska and Szymańska [35] also noted that rural areas, especially those located around large cities, continue to accept more new residents, most of whom are working-age adults. These demographic dynamics contribute to revitalizing the demographic structure of these areas and indirectly strengthening the economic bases there.

According to Báčová and Krása [47], fewer people in the agriculture sector could be related to the use of heavier and larger machines, which in turn results in less local focus, less land observation, and less small-scale measures. 

Poverty can be considered one of the reasons for the increase in soil erosion rates, mainly due to wrong decisions being made about land management [48]. It is well documented (see, e.g., [2,7,8,12,13]) that the income of families working on agricultural land is greatly affected by the soil erosion rate because accelerated soil erosion causes nutrients on the soil surface to wash away and the soil to lose its production capacity. Farming households will, in turn, have to use more chemical fertilizers or livestock to maintain and even increase soil production capacity, although this is unlikely to be sustainable in the long run. Furthermore, applying chemical fertilizers, as agricultural input, will reduce the final profit of farmers. 

Optimal soil management [2,12] is a decision-making task that deals with the preferences and priorities of farmers and has long-term biophysical and economic consequences. Therefore, the selection and implementation of land management strategies affect the amount of soil erosion. The income of farmers can hence be considered as a potential factor that affects their preferences for managing or using land resources. Therefore, land-conservation strategies favor those strategies in which land degradation, in general, and soil erosion, in particular, decrease as the average household income increases.

More specifically, Eaton [8] showed that the initial and ongoing costs of alternative cropping systems, which can include additional inputs and maintenance costs, affect soil erosion rates because land users, such as farmers and ranchers, are always seeking to reduce production costs so that they can increase their marginal profits. Therefore, decision-making when choosing the type of crop or planting method can be easier if those decisions can improve farmers’ incomes.

Soil can be considered a potentially profitable asset whose protection requires direct costs, including labor, materials, and equipment. These costs are borne primarily by farming and ranching households, consequently reducing their marginal profits. The direct costs of conserving the soil, regardless of the medium- and long-term benefits that it can bring, will certainly reduce the marginal benefits of these households in the short term. Consequently, different behaviors can be expected with respect to the adoption of land conservation strategies, considering household income. In this regard, Barbier [13] showed that some farmers may allocate part of the household budget to soil conservation measures, while others might be reluctant to accept the direct costs that can be incurred. Other households might apply planting systems that have an economic advantage despite increasing soil erosion rates [18].

However, the area-weighted average income per capita variable did not enter the models developed using the stepwise regression approach. Otherwise, due to the integration of income per capita in the calculation of the human development index, it was most likely that the models would be exposed to multicollinearity drawbacks.

Several studies (see, e.g., [12,19,49]) revealed that soil erosion results in a decrease in GDP. If the share of the agricultural sector in GDP is high, soil erosion can have very significant effects on GDP.

The question that may arise is whether economic prosperity causes environmental degradation. In other words, do affluent people deal with land resources, thereby causing environmental degradation or improving the state of the environment? In this regard, although studies on the influences of soil erosion on GDP have been conducted at the macroeconomic level (e.g., see [12,21]) and sometimes on a global scale [9], the spatial analysis of change in GDP in relation to the soil erosion rate shows that increasing GDP is associated with reducing soil erosion rates in the study catchments (Equations (2), (4) and (5)).

The question now is whether economic well-being, which can be measured by the Human Development Index, causes the destruction of the environment or improves it. Economic well-being is usually measured by real amounts of income or the real value of GDP. However, HDI was introduced to measure economic well-being at different administrative levels, ranging from zero for complete dissatisfaction with life to one for complete satisfaction. The results of this study (Equations (2)–(5)) show that if economic well-being increases, the soil erosion rates decrease in the study catchments. 

### 3.2. Results of the Goodness of Fit of the Models

**Figure 2:**
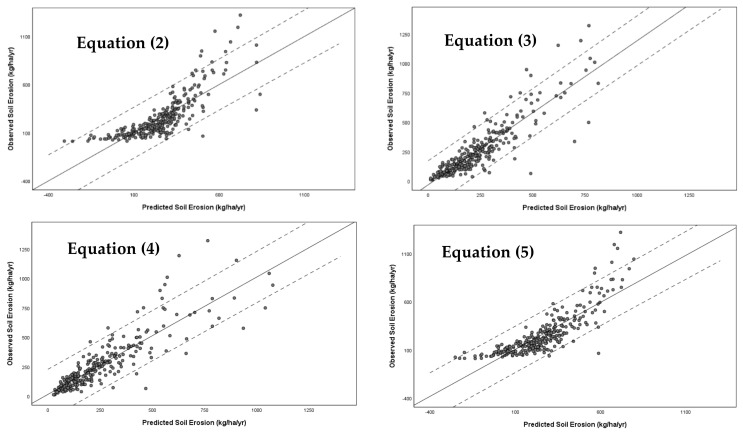
One-to-one relationship between predicted and observed values.

The goodness of fit of the developed models (Equations (2)–(5)) was examined referring to the value of the coefficient of determination, the significance of the model and its coefficients at the level *p* < 0.05, and the collinearity of the independent variables of the models based on the variance inflation factor. Figure 2 shows the predicted values vs. the observed values. Table 2 shows the statistics of the coefficient of determination, the significance coefficients of the models, and the bias coefficient of variance.

### 3.3. Results of the Inter-Model Comparison

The results of the Akaike information criterion are given in Table 3. The comparison shows that of the four models developed, Equation (3) can be selected as the most appropriate.

### 3.4. Results of the Model Sensitivity Analysis

The appropriate model (Equation (3)), based on the results of the Akaike information criterion, was selected to perform the sensitivity analysis. The result of the sensitivity analysis is shown in Table 4, which is based on the slope of the line that shows the relationship between changes in soil erosion variables due to changes in the values of independent variables. Consequently, among soil factors, soil-erosion rates are the most sensitive to changes in soil organic carbon content. The slope of the terrain is ranked fourth. The population density is comparatively ranked as the second variable; the model response is sensitive to it (Table 4). 

### 3.5. Behavioral Comparison of Soil Erosion in Relation to Socio-Economic Variables

The analysis of soil erosion behavior in relation to changes in socio-economic variables was carried out using a procedure called incremental analysis, which is inspired by conditional sensitivity analysis. Consequently, incremental changes in the model response are calculated by incrementally changing the values of the independent variable, fixing the values of other independent variables with their means.

**Figure 3:**
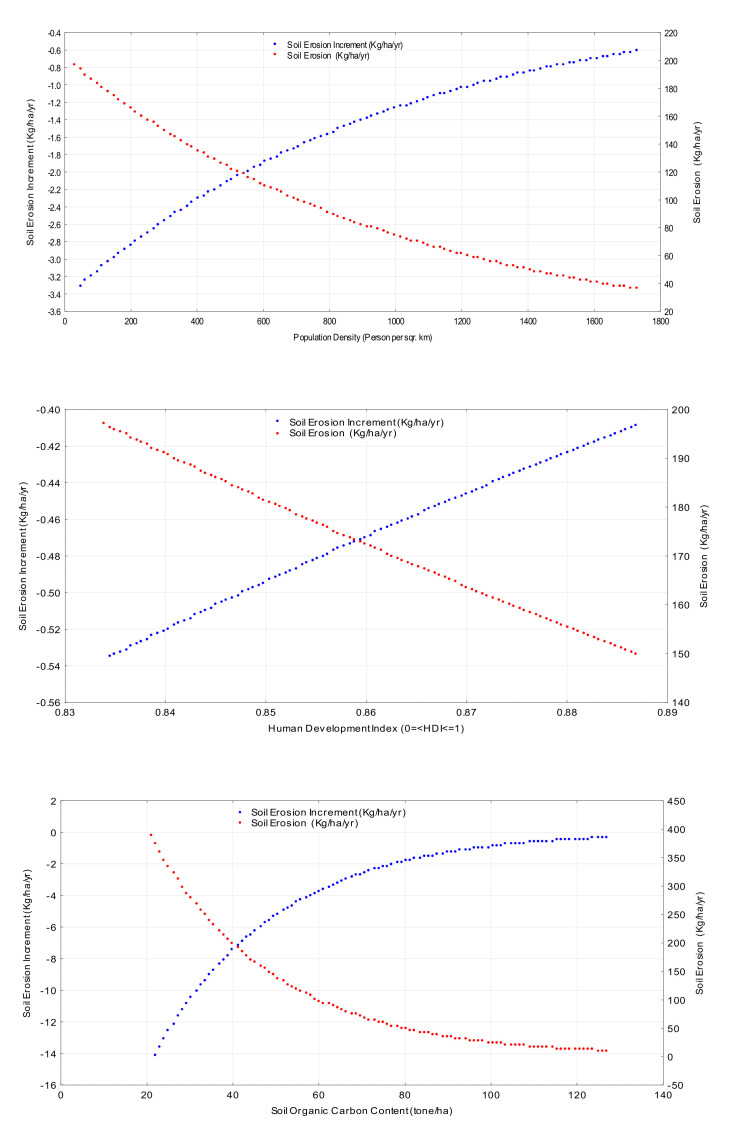

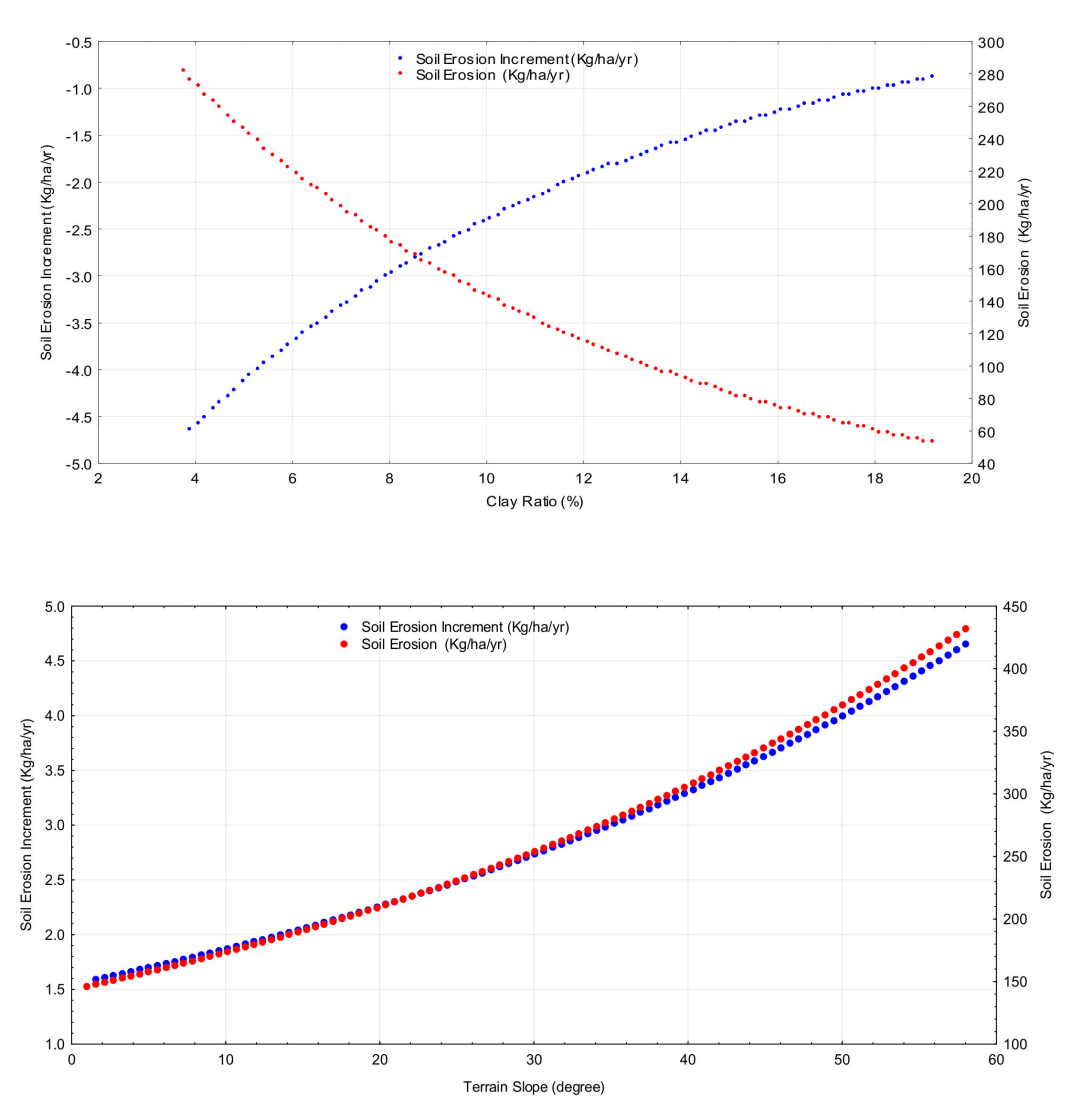
Incremental effect analysis of the soil-erosion model to independent variables.

The plot of the incremental values of soil erosion versus the incremental changes in the values of the variable of interest (Figure 3) shows that the soil-erosion model does not have a uniform response by changing the values of the independent variables. 

## 4. Conclusions

In this study, we found that socio-economic factors have significant effects on the amount of soil erosion and can be used as factors driving the rate of soil erosion in the study catchments. 

The inverse relationship between GDP and soil erosion rates implicitly indicates that economic well-being leads to correct decisions in favor of conserving landscapes. Therefore, increasing society’s economic well-being reduces the degree of landscape degradation, in general, and the rate of soil erosion, in particular. It should be noted that the opportunity to improve the environmental quality that economic prosperity provides in the shadow of increasing GDP is applicable only to man-made environments, such as cities and urban areas. Lost opportunities, such as the extinction of plant and animal species due to the degradation of natural landscapes, cannot be compensated for through the opportunities that economic advances provide later on.

Increasing the value of the human development index is associated with reducing land degradation and reducing the amount of soil erosion. Taking into account the components of HDI, which include a long and healthy life, knowledge, and a decent standard of living, it can be stated that improving living conditions such as health, education, living standards and human perceptions, as well as people’s awareness of the long-term benefits of implementing landscape degradation control plans, directly and indirectly, affect soil erosion rates.

## Figures and Tables

**Table 1:** Area (km^2^) statistics for the study catchments in Poland [38].

Data Layers (Pfafstetter Level)	No. of Catchment	Mean	Min.	Max.	Sd.	Variance
3	1	696,040	-	-	-	-
4	5	71,664	427	193,306	73,407	5,388,614,924
5	21	17,063	102	84,920	20,527	421,348,626
6	42	7937	21	33,314	8222	67,601,246
7	140	2358	18	14,168	2276	5,182,054
8	443	700	0	3687	640	410,207
9	1268	246	0	1452	197	38,965
10	2240	139	0	665	75	5559
11	2429	129	0	325	129	16,528
12	2430	129	0	325	59	3434
The study catchments	402	662	0.23	2758	615	378,305

**Table 2:** Statistics of regression models for soil erosion rate in the study catchments.

Model No.	Model Variable	Coefficients						Collinearity Statistics	
		B	Std. Error	Beta	*r^2^*	*t*	*p-*Value	Tolerance	VIF
2	Constant	2410.252	531.197		0.64	4.537	0.000		
	SOC	−8.366	0.792	−0.587		−10.562	0.000	0.421	2.374
	Slp	10.741	1.085	0.374		9.904	0.000	0.914	1.094
	CR	−19.547	4.271	−0.232		−4.577	0.000	0.504	1.983
	Stg	−0.461	0.113	−0.178		−4.064	0.000	0.675	1.482
	GDP	−0.538	0.160	−0.124		−3.357	0.001	0.952	1.051
	HDI	−2039.122	618.735	−0.122		−3.296	0.001	0.949	1.054
3 *	Constant	11.814	1.581		0.791	7.474	0.000		
	SOC	−0.035	0.002	−0.634		−17.966	0.000	0.605	1.654
	CR	−0.108	0.012	−0.330		−9.238	0.000	0.592	1.690
	Slp	0.019	0.003	0.172		6.079	0.000	0.941	1.063
	PoD	−0.001	0.000	−0.115		−4.006	0.000	0.917	1.090
	HDI	−5.138	1.844	−0.079		−2.787	0.006	0.940	1.064
4	Constant	11.981	0.430		0.783	27.895	0.000		
	SOC	−1.662	0.107	−0.631		−15.583	0.000	0.480	2.083
	CR	−0.915	0.111	−0.335		−8.243	0.000	0.476	2.099
	Stg	0.290	0.043	0.192		6.795	0.000	0.984	1.017
	HDI	−5.485	1.563	−0.101		−3.509	0.001	0.949	1.054
	GDP	−0.133	0.044	−0.090		−3.034	0.003	0.895	1.118
5	Constant	1828.741	129.764		0.707	14.093	0.000		
	SOC	−388.269	32.972	−0.559		−11.776	0.000	0.472	2.119
	Slp	127.096	13.443	0.319		9.455	0.000	0.937	1.067
	CR	−237.037	34.177	−0.329		−6.936	0.000	0.472	2.119
	GDP	−50.363	13.327	−0.130		−3.779	0.000	0.904	1.106
	HDI	−1413.787	486.447	−0.099		−2.906	0.004	0.920	1.087

* The most appropriate model.

**Table 3:** Results of the inter-model comparison using the Akaike information criterion for soil-erosion regression models.

Model No.	RSS	*n*	Log (RSS/*n*)	K	2 K	K + 1	*n* − K − 1	AIC	Δj	EXP (−0.5 × Δj)	Wi
2	15,254,988.25	284	4.73	7	14	8	276	1357.35	6.99	0.03	0.03
3 *	14,650,604.57	284	4.71	6	12	7	277	1350.36	0	1	0.97
4	17,185,967.99	284	4.78	6	12	7	277	1370.05	19.69	5.3106 × 10^−5^	0.00
5	16,171,040.85	284	4.76	6	12	7	277	1362.54	12.18	0.00	0.00

* The most appropriate model.

**Table 4:** Results of the sensitivity analysis of the selected soil-erosion model.

Variable Name	Formula	Rank
Soil organic carbon content	Y = 4.1 – 1.09x	1
Clay ratio	Y = 4.81 – 0.49x	3
Terrain slope	Y = 5.53 + 0.32x	4
Human Development Index	Y = 5.15 – 0.08x	5
Population density	Y = 4.43 – 0.5x	2

## Data Availability

All data are available upon request.
